# A comparison of population estimation techniques for individually unidentifiable free-roaming dogs

**DOI:** 10.1186/s12917-019-1938-1

**Published:** 2019-06-07

**Authors:** N. V. Meunier, A. D. Gibson, J. Corfmat, S. Mazeri, I. G. Handel, L. Gamble, B Mde C Bronsvoort, R. J. Mellanby

**Affiliations:** 1 The Royal (Dick) School of Veterinary Studies (R(D)SVS) and the Roslin Institute, Hospital for Small Animals, Easter Bush Veterinary Centre, Midlothian, EH25 9RG Scotland; 2 Mission Rabies, 4 Castle Street, Cranborne, Dorset, BH21 5PZ UK; 3 The Epidemiology, Economics and Risk Assessment (EERA) Group, The Roslin Institute and the Royal (Dick) School of Veterinary Studies (R(D)SVS), Easter Bush, Midlothian, EH25 9RG Scotland

**Keywords:** Population estimation, Dog abundance, Free-roaming dogs, Mark resight

## Abstract

**Background:**

Measuring the size of free roaming dog populations quickly and accurately is critical in the implementation of numerous preventive health and population control interventions. However, few studies have investigated the relative performance of population size assessment tools when applied to dogs. The aim of this study was to compare the commonly used mark-resight methodology with distance sampling methods, which are less resource intensive, to estimate free-roaming dog abundance in Goa, India.

Twenty-six working zones were surveyed along all roads twice by the same surveyor at the same time of day, following a vaccination campaign which included marking of vaccinated dogs with a coloured paint. The Chapman estimate was then used to evaluate the mark-resight abundance. Additionally, the number of dogs and perpendicular distance from the road for all dogs sighted was recorded. This was used to estimate dog density and abundance using distance sampling methods. The detection function was fitted based on goodness-of-fit and AIC.

**Results:**

The Chapman abundance estimate for the entire study area was 5202 dogs (95%CI 4733.8–5671.0), and the distance sampling method abundance estimate was 5067 dogs (95%CI 4454.3–5764.2). For individual working zones, after taking other factors into account in a mixed effects model, the average distance sampling estimate was 35% higher (95%CI 20–53%) than the Chapman estimate. There was also evidence of a difference in estimates between surveyors of 21% (95%CI 7–37%) and between days (22% lower on day 2, 95%CI 8–38%) for individual working zones.

**Conclusion:**

Our study demonstrated that the distance sampling estimates were comparable overall to the Chapman method of abundance estimation of free roaming dogs across the entire study region but there was noticeable variation between the two methods when individual zones were compared. Consequently, distance sampling methods may be suitable to enumerate dogs over large areas in a more time efficient manner than the widely used mark-resight approach.

**Electronic supplementary material:**

The online version of this article (10.1186/s12917-019-1938-1) contains supplementary material, which is available to authorized users.

## Background

Domestic dogs are the source of almost all human rabies cases [1], but mass vaccination of dogs has been shown to dramatically reduce canine rabies, and subsequently human cases [2]. In addition to mass vaccination, the World Health Organisation recommends animal birth control programmes to reduce stray dog populations [3, 4]. The vaccination coverage target is accepted to be 70%, which was modelled as effective to control the transmission of the rabies virus [5], with many campaigns meeting this target [6]. However, the main challenge in vaccination campaigns is to meet a sufficient vaccination coverage of the dog population [7] and to measure this impact. In order to successfully implement these vaccination programmes, there is a clear need to be able to estimate the size of dog populations quickly and accurately.

The World Society for the Protection of Animals, and the Rabies Blueprint produce survey guidelines for measuring the abundance of dogs and vaccination coverage after a rabies vaccination campaign [8, 9]. These include door-to-door surveys, sampling, indicator counts, and capture-mark-recapture methods. Methods looking at absolute abundance are time and resource intensive and this can limit their use for regular population studies [10]. Indicators, such as repeated direct counts along prescribed routes [11], are more feasible for longitudinal studies, but give no indication of total abundance. Enumerating dogs is important for planning operational activities at the start of a vaccination or sterilisation campaign, and serve as a benchmark for population interventions. Additionally, abundance estimates can be used to calculate vaccination coverage as a measure of effectiveness of the interventions.

Free-roaming dogs, i.e. dogs that are not restricted in their movements, can be difficult to enumerate if they are unowned and traditional door-to-door surveys may not accurately account for these dogs. Household surveys are therefore effective in certain countries such as Tanzania, Malawi, and Chad with lower unowned populations but may be less suitable for areas with a significant unowned, free-roaming population such as in India [1, 6, 12]. Free-roaming dogs have been described as difficult to catch and vaccinate, serving as a reservoir for rabies [7]. Understanding, this free-roaming population is therefore important for the control of infectious diseases [13].

Good enumeration techniques are available in ecological studies and ideally involve identification of individual animals [14]. Arguably, the most commonly used approaches are variations on the capture-mark-recapture methodology, for which multiple analysis techniques have been developed. This involves initially capturing and marking animals, which can be either physically restrained and marked with, for example, dyes, collars or ear-notching, or photographic capture, as was pioneered in stray dogs by Beck (1973) [15]. Animals are then released and recaptured or resighted on a second occasion, with identification of the marks. Individual identification can be resource intensive e.g. photographic comparisons [16, 17] and not well suited to the operational practicalities of mass vaccination campaigns, especially with a large unowned proportion. Additionally, physical marking of stray animals may not be possible without the large resources required for capture.

Distance sampling techniques do not require capture or marking of animals. These rely on measuring the perpendicular distance of the animal from randomly placed transects through the study area. These methods have been infrequently used for dog population estimation but have been reported in the Philippines [18]. Our study wished to explore a simple distance sampling technique for estimating the abundance of free-roaming dogs when individual animals could not be identified.

The aim of this study was to compare the frequently used mark-resight methodology against a less time and resource intensive distance sampling technique to enumerate the size of free roaming dog populations in regions of Goa, India. Secondly, we wished to investigate if surveys using a subset of roads for these estimations would give similar results.

## Materials and methods

### Study site

**Fig. 1:**
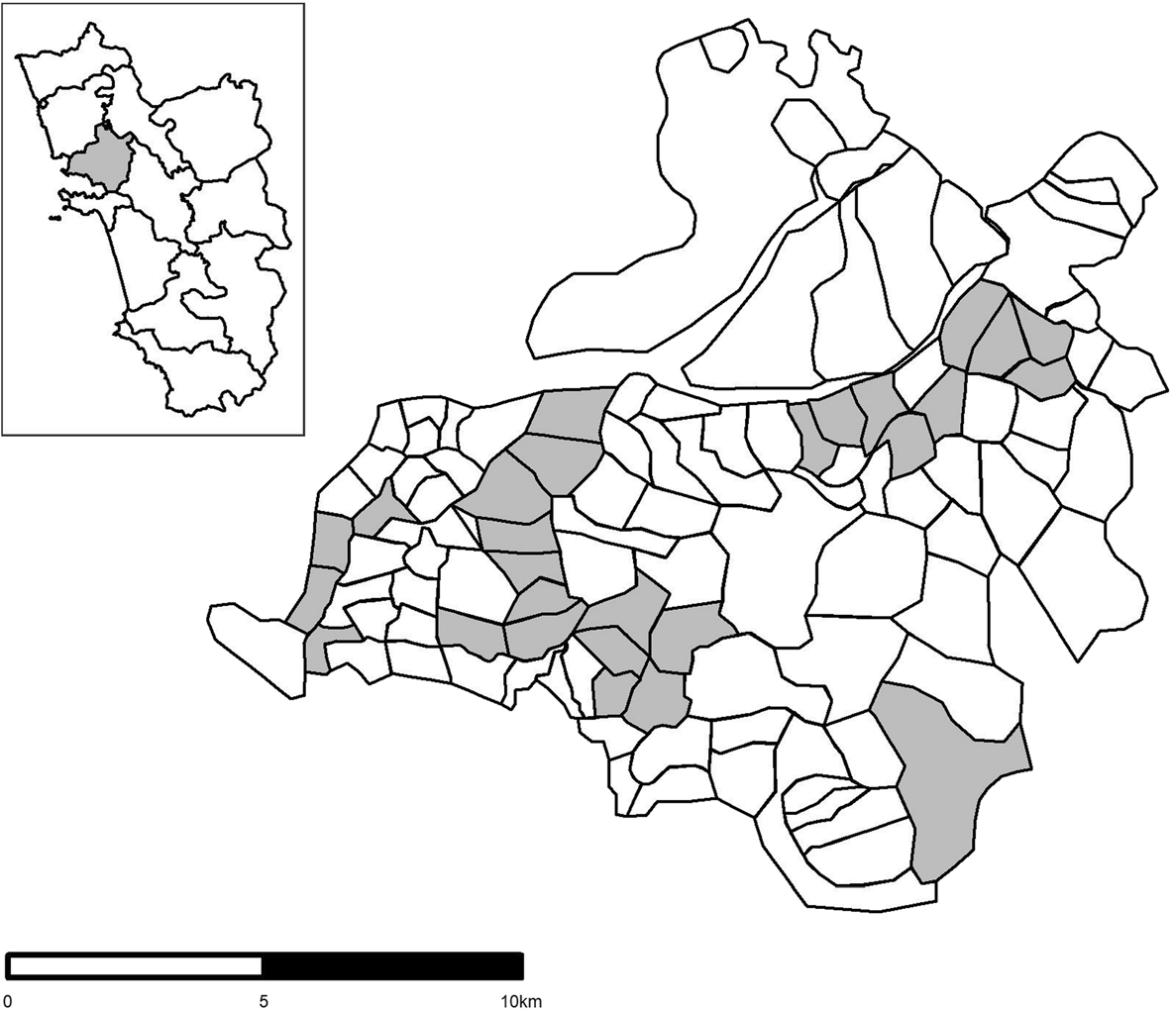
(Inset) Map of Goa, showing Tiswadi taluka in grey, (Main) Tiswadi taluka showing working zones highlighted in grey (*n* = 26)

The study took place in the Tiswadi taluka of the state of Goa in south west India, following a vaccination project by the Mission Rabies charity during March and April 2018 (Fig. 1). Each panchayat (ward) of the region was further subdivided, based on housing density, into smaller working zones which could be covered by a vaccination or survey team within one to three days. Goa administrative boundary data were obtained from GADM (https://gadm.org/data.html). All working zones in which vaccination activities occurred during the study period were considered for inclusion in the study. These were selected based on the logistics of the campaign and were considered typical for the region. The study area covered a range of settlement types from rural villages to urban areas. The average population and number of households per panchayat included in the study were not statistically different to all panchayats in Goa. Dogs were classified as free-roaming if they were seen on the streets or were in yards with access to the street. Dogs within houses, behind closed gates, or tied within a property, were considered confined and were outside the scope of this study.

### Marking methods

Vaccination teams of 5–8 individuals, were allocated working zones and instructed to vaccinate all dogs within the demarcated area covering all accessible roads. Each rabies vaccination was recorded on a designated smartphone app (WVS Data collection App), capturing details on sex, age, confinement status, and GPS location [19, 20]. Dogs were marked with a coloured paint before release. Free-roaming dogs were captured by hand or by net, depending on their temperament. After completion of a working zone by one team, the area was revisited, either on the same day or the following day, by another vaccination team in an attempt to vaccinate any missed dogs.

### Survey techniques

Sight surveys were conducted in each working zone within 48 h after vaccination. Surveys were conducted by a single surveyor travelling by moped, instructed to travel down every street of the designated working zone to sight dogs. Surveys were completed either in the morning or afternoon, and were repeated by the same surveyor, at the same time on the following day, following the same routes. For each sighted dog, the surveyor entered the sex, age, and mark status (paint mark present or absent) of a dog into the smart phone app, which also recorded the GPS location. Only free-roaming dogs were recorded. Additionally, the surveyor estimated the perpendicular distance of the dogs from the centre of the road, as well as the road width. Surveyors were trained before the study to estimate distances, with weekly refreshers during the study period.

**Fig. 2:**
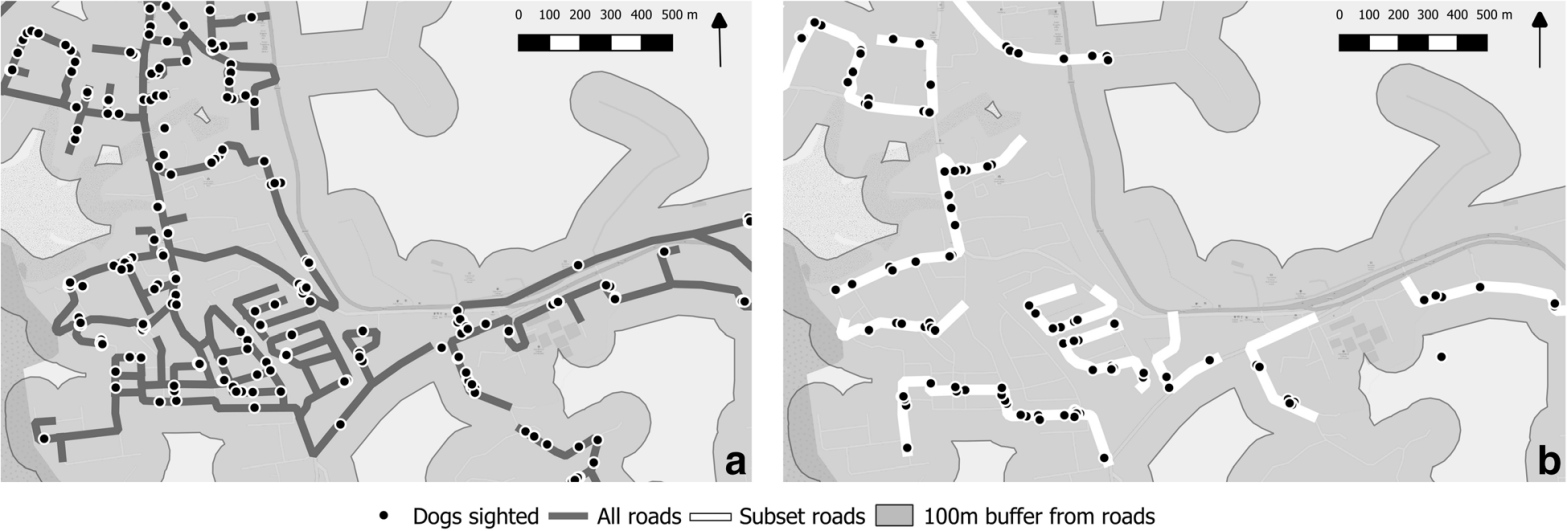
Close-up map of a typical study area, showing routes covered during the survey **a**) all roads, **b**) subset roads, dogs sighted (black points), and the study area buffer within 100 m of roads (grey shading). Dogs were not expected to be seen outside of the buffer areas. Adapted from OpenStreetMap

At the same time as the main survey, another surveyor sighted dogs along a pre-specified subset of roads in the same area, at the same time of day. This was done for the purpose of evaluating the agreement in the estimates between the standard estimation techniques along all roads, and using a subset of roads which would be less resource and time intensive. The subset of roads was available on a map to the surveyors via the smart phone app (Fig. 2). These were selected before the study by attempting to select roads along a systematic zig-zag pattern per area based on the OpenStreetMap [21] road vector map. This included main roads as well as smaller residential roads and paths. Surveyors followed a rota to switch between surveying all roads or the subset, so that both techniques would be covered by all surveyors.

The 26 working zones covered an area of 20.94 km^2^ within 100 m of the roads network excluding forested or farming land. The road network covered 100.91 km and the subset of roads covered a network of 47.20 km of roads. Each work zone was surveyed 4 times (all roads and a subset of roads, repeated the following day) with a total of 104 surveys. Two trained surveyors covered the bulk of the surveys with an additional trained surveyor conducting 4 surveys due to staff illness. A further 11 work zones were surveyed but not included in the study due to incomplete surveys or repeated surveys not conducted because of staff illness or redeployment, or because data were lost owing to technical difficulties on the smartphones.

### Analysis

#### Population estimation

The sight surveys were analysed using the mark-resight Chapman estimate, to estimate dog abundance, taking the proportion of marked and unmarked dogs into account. The estimates were calculated for each survey per work area and totalled across all areas. Averages of the two surveys per area were also calculated.

The Chapman estimate is given by [22]:

$$ N=\left[\frac{\left({n}_1+1\right)\left({n}_2+1\right)}{\left(m+1\right)}\right]-1 $$

Where *N* is the population size being estimated, *n*_1_ is the number of animals initially marked, *n*_2_ is the number of animals sighted during the follow-up survey, and *m* is the number of marked animals sighted in the follow-up survey.

The perpendicular distances of dogs to the roads were analysed in R with the package *Distance* [23], to estimate, firstly, dog density per area and secondly, dog population abundance. The transect line was considered to be 1 m inside the road edge and the perpendicular distance measured to this line. Any dogs in the centre of the road were assigned as being on this transect line. The centre of the road was usually avoided by dogs due to car traffic, and they tended to rest along the road edge. The detection function models for the distance sampling method were selected by AIC, after plausible selection on Chi-square goodness-of-fit tests and visualising the function shape, with varying adjustment terms (cosine, simple polynomial, hermite polynomial). Distances measured were grouped in 5 m categories centred on multiples of five to avoid increased frequencies of easily reported numbers. The truncated distance was 37.5 m. The final detection function model key functions were half-normal with cosine adjustments for the data from all roads, and hazard-rate with independent variables for the subset road data. The final models are listed in the additional materials (Additional file 1: Table S1).

The area used to estimate the abundance was restricted to within 100 m of a road. This was calculated in QGIS [24] by adding a 100 m buffer around all known roads from a OpenStreetMap vector map of the area [21], to calculate reasonably likely areas (in metres squared) where dogs would be found. This was confirmed on satellite image overlay to exclude forested areas or fields but include all built up areas.

#### Comparative analysis

A comparative analysis was firstly conducted of the numerical estimates for the dog population abundance between the Chapman and distance sampling methods. Secondly, we compared the estimates between using all roads and the subset of roads.

Lin’s Concordance Coefficient was calculated for the comparisons and Bland-Altman plots (Additional file 2: Figure S1) allowing visualisation of the patterns of agreement between the methods [25, 26].

A mixed-effects regression model was used to analyse factors associated with the estimated abundance. The calculated abundance estimate was log transformed as the outcome measure for the final mixed-effects model. The final fixed effects were the type of estimate (Chapman or distance-method), surveyor, first or second survey, all road or subset survey, and time of day. The work zone was considered a random effect. No interactions were selected for the final model. The final mixed model was selected based on AIC and the residuals were visually assessed.

## Results

### Comparison at study level

A summary of the abundance estimates for the 26 work zones evaluated are given in Table 1, divided into surveys for all roads and the subset roads, as well as the first and second survey. For the surveys that covered all roads, the standard technique, the average proportion of marked dogs was 0.45 (SD 0.22) with an average of 23 dogs (SD 10) seen per area. The Chapman abundance estimate for the entire study area considering data from all roads was 5202 dogs (95%CI 4733.8–5671.0), and the distance sampling method estimate was 5067 dogs (95%CI 4454.3–5764.2). The density estimate from the *Distance* software was 242 dogs per km^2^ (95%CI 213–275) for all roads as transects, and an average of 6 dogs (SD 1.9) were seen per km of road covered.

**Table 1:** Summary of surveys giving mean mark proportion per working zone, mean number of dogs sighted per working zone and total across areas, Chapman and distance sampling method estimates with 95% confidence intervals. In bold: Means of repeated surveys, abundance estimates calculated with data from both surveys

	Mark proportion		Number of dogs sighted			Chapman abundance		Distance-method abundance	
	Per working zone		Total area	Per working zone		Total study area		Total study area	
	Mean	(SD)		Mean	(SD)	Estimate	(95% CI)	Estimate	(95% CI)
All roads									
Both surveys	**0.45**	**(0.22)**		**22.63**	**(9.70)**	**5202.36**	**(4733.75–5670.97)**	**5067.06**	**(4454.27–5764.17)**
Survey 1	0.41	(0.25)	559	21.42	(9.27)	6438.73	(6044.40–6833.07)	5681.90	(4945.60–6527.81)
Survey 2	0.48	(0.18)	622	23.84	(10.15)	3965.99	(3683.61–4248.36)	4948.98	(4296.05–5701.14)
Subset roads									
Both surveys	**0.41**	**(0.23)**		**13.65**	**(5.83)**	**5190.76**	**(4491.27–5890.26)**	**5810.61**	**(5141.12–6567.28)**
Survey 1	0.40	(0.24)	359	13.77	(5.02)	5743.77	(5239.96–6247.59)	7019.16	(5529.45–8910.22)
Survey 2	0.42	(0.22)	358	13.53	(6.64)	4637.75	(4157.64–5117.87)	5295.03	(4385.32–6393.45)

**Fig. 3:**
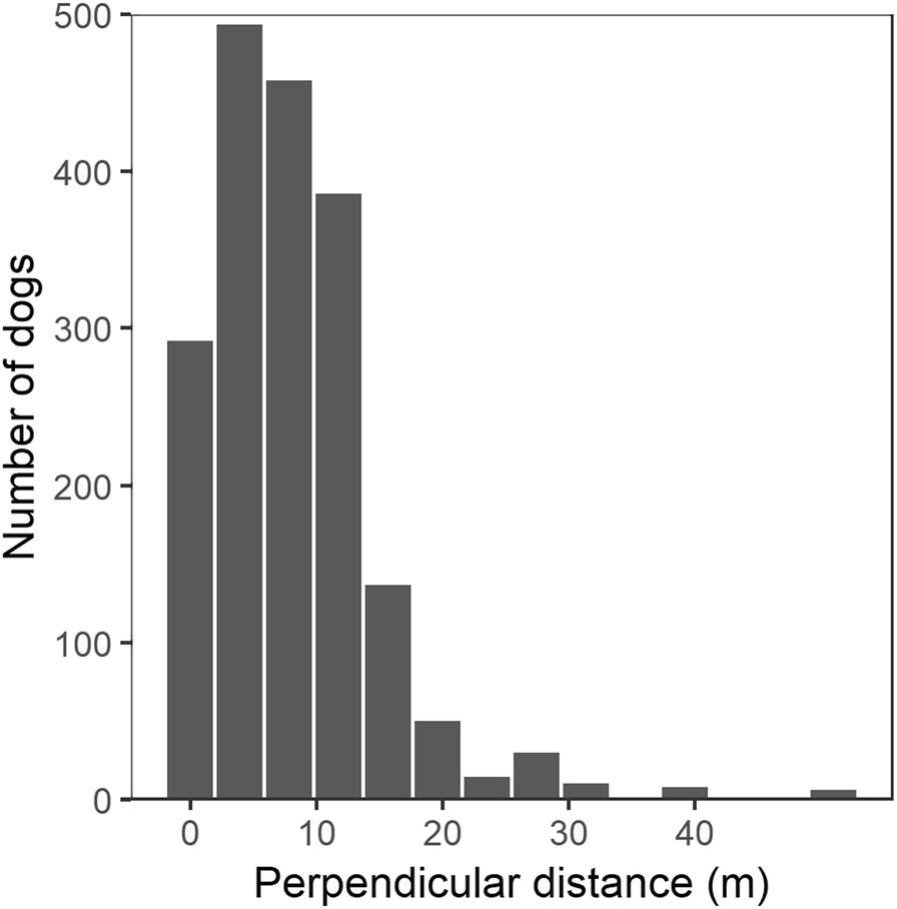
Histogram of the perpendicular distance a dog was sighted away from the road

For the distance sampling method estimates, the perpendicular distance of a dog from the road edge (transect) was measured. These distances are summarised in Fig. 3, with the majority of distances being less than 20 m from a road edge. Increased frequency of values centred on multiples of 5 can be seen due to the estimates made by the surveyors and the data were therefore categorised for selecting the detection function. Anecdotal evidence suggested that housing, walls, and dense bush often prevented detecting dogs beyond this 20 m distance.

### Comparison at work zone level

**Fig. 4:**
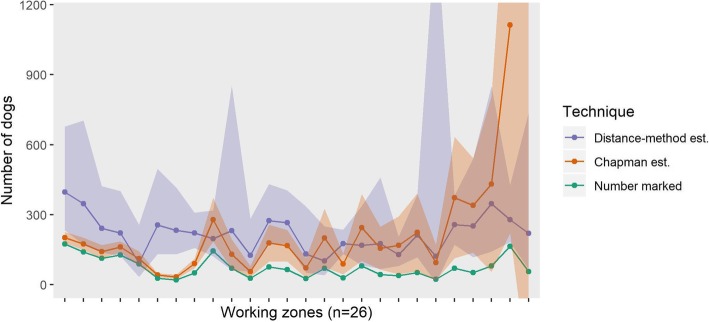
Number of dogs estimated per working zone ordered in decreasing marking proportion. Confidence intervals (shaded area) for the distance sampling method estimate (circle) and Chapman mark-resight estimate (triangle). The number of dogs initially marked (vaccinated) per area (diamond) is also given

The abundance estimates are visualised per work zone in Fig. 4, plotted against the number of dogs vaccinated per area for the first all road survey. The order from left to right is in decreasing proportion of marked dogs sighted and as the marked proportion decreases, the Chapman confidence intervals become larger.

**Fig. 5:**
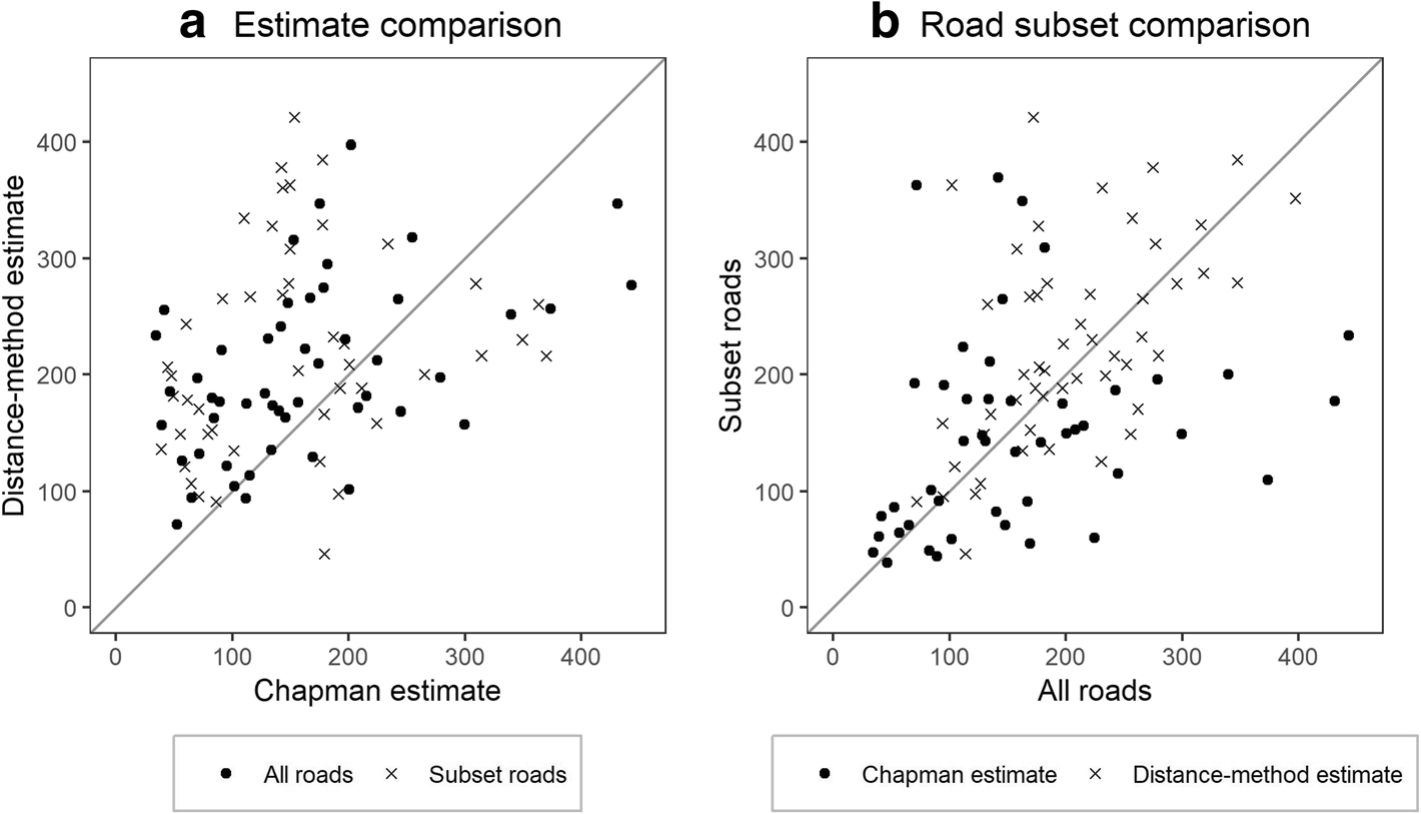
Plot comparing, **a**) Chapman vs distance sampling method estimates, and **b**) estimates from all roads vs subset of roads

On average, the Chapman abundance estimate was 20.76 lower (95%CI -58.53, 17.01) than the distance sampling method estimate, which can be seen visually in the skewness of the scatterplot between these measures (Fig. 5a). Lin’s concordance correlation coefficient (CCC) was 0.17, which indicates a poor agreement between these measures at the work zone level without adjustment.

From the mixed-effects model, the expected dog abundance estimate per working zone for the first morning, all-road survey using the Chapman method was 158 dogs (Table 2). The distance sampling method estimate was 35% higher (95%CI 20–53%) than the Chapman estimate taking the surveyors, survey order, survey type and time of day into account. With the sampling method used as a co-factor in the model, there was also evidence of a difference between surveyors of 21% (95%CI 7–37%) and between days (22% lower on day 2, 95%CI 8–38%). There was no evidence of a difference between all road and subset road surveys, or surveys conducted in the morning or afternoon.

**Table 2:** Estimates from the mixed effects model, showing fixed and random effects, as well as the intraclass correlation coefficient (ICC) for the random effects. The log transformed abundance estimate was modelled as the outcome. Coefficients are exponentiated and *p*-values given

Fixed effects	EXP(β)	95% CI	P-value
Intercept	157.83	(125.47–198.41)	< 0.001
Distance-method vs Chapman	1.35	(1.20–1.53)	< 0.001
Surveyor B vs A	1.21	(1.07–1.37)	< 0.01
Survey 2 vs survey1	0.82	(0.72–0.92)	< 0.01
Subset vs all roads	1.05	(0.92–1.19)	0.48
Afternoon vs morning	0.80	(0.57–1.11)	0.19
Random effects	Variance	Std. Dev.	ICC
Working zones	0.14	0.38	0.41
Residual	0.20	0.45	

### Comparison of full survey versus subset of roads

In the surveys that only covered a subset of roads, the mean marked proportion of dogs was 0.41 (SD 0.23), with an average of 14 dogs (SD 6) per survey. The abundance estimate for the Chapman technique for the study area considering only the data from the surveys covering the subset of roads was 5191 dogs (95%CI 4491.3–5890.3), with the distance sampling method estimating 5811 dogs (95%CI 5141.1–6567.3). The density estimate from the *Distance* software was 277 dogs per km^2^ (95%CI 245–314) for the subset of roads, and 8 dogs (SD 2.7) were seen on average per km of road covered.

When comparing the surveys that covered all roads versus those that only covered the subset of roads, the Chapman estimate mean difference was 0.45 (95%CI -56.96, 57.85) and the CCC was 0.41 indicating moderate agreement. There was marked variation at higher estimates as seen in Fig. 5b. For the distance sampling method estimates, the mean difference was 32 dogs fewer (95%CI -61.83, − 2.91) for all roads compared to the subset surveys, and the CCC was 0.37 (Table 3).

**Table 3:** Method comparison showing the mean difference between methods, Lin’s Concordance Correlation Coefficient (CCC), and Pearson’s correlation coefficient. The abundance estimates between the Chapman and distance sampling methods is compared; as well as the abundance estimates, marked proportion seen, and number of dogs counted per survey for the comparison of all roads to the subset

Comparison	Mean difference		Lin’s CCC		Pearson’s R	
Chapman vs distance-method	−20.76	(−58.53, 17.01)	0.17	(0.01, 0.31)	0.21	(0.01, 0.38)
All roads vs subset						
Chapman estimate	0.45	(−56.96, 57.85)	0.41	(0.17, 0.61)	0.43	(0.17, 0.63)
Distance-method estimate	−32.37	(−61.83, −2.91)	0.37	(0.15, 0.55)	0.43	(0.18, 0.63)
Marked proportion	0.03	(−0.06, 0.13)	−0.12	(− 0.37, 0.16)	−0.12	(− 0.38, 0.16)
Number of dogs	8.98	(6.84, 11.12)	0.33	(0.18, 0.46)	0.61	(0.40, 0.76)

## Discussion

Across the total study area, the distance sampling method gave comparable abundance estimates to the Chapman method. This supports the possibility that distance sampling may prove to be a cost and resource efficient method for estimating free-roaming dog populations but requires more studies over larger areas. There was variation at the working zone level but many areas still had overlapping confidence intervals between methods (Fig. 4). While some of this variation can be explained by differences in surveyors, these differences may also be due to expected natural variability in dog sightings.

Belo et al., (2017) observed that individual census counts do not adequately estimate abundance, as there are differences in dog detection [27]. The number of dogs counted in each working zone was small on average, and the proportion of marked dogs and corresponding Chapman estimate, would be heavily influenced by this, which is a major limitation of this study. It is also not known for how long the initial disturbance of the capture operation will influence the resident dog population to disperse out of the area. At the working zone level, there was evidence that the distance sampling method estimates were higher compared to the Chapman estimate when adjusting for surveyors, surveys, time and type of survey. This was more noticeable when looking only at the subset of roads. There was no evidence from the statistical model of a difference between the estimates from all roads versus the subset of roads, although the subset of roads produced more uncertainty. The actual number of dogs counted per survey was low compared to the estimates, and direct counts for the purpose of a census would not be recommended, except as an indicator count.

This study estimated an average dog density of 242 dogs per km^2^ (95%CI 213–275) which was higher than Mumbai, 57 dogs per km^2^ [28], and Katwa, 178 dogs per km^2^ [29], and within the range of densities reported in other countries [14]. The higher densities reported here compared to other Indian studies may be due to this study limiting the study area to within 100 m of the road network.

Considering the density of dogs per km of road, this study reported an average of 6 dogs (SD 1.9) per km of road covered, which was lower than a study in Nepal, but higher than Latin American estimates [30]. Again, these estimates are highly variable dependant on location. A higher density of dogs was seen in town neighbourhoods compared to rural areas in a Bhutan study [22], and this variation between studies may be accounted for by housing density, geographic features, land use, or survey method [14].

Assumptions for the mark-resight method used here, include a closed population and no mark loss. Resight surveys were conducted within 48 h of the vaccination and marking. This short time interval should allow for minimal population changes for the closed population assumption to be valid. While dogs generally stay near their homes, some dogs can have large home ranges [31] and the borders of our working zones were arbitrary. Movement into and out of the study areas is therefore possible but was difficult to measure. This study did not account for mark loss due to the short time interval of less than 5 days, however it was possible for misclassification to occur. Conan et al., (2015) saw difficulties with darkly coloured dogs as well as misclassification of colours [32]. Additionally, dogs may have been double-counted within the same survey if they moved, as individual identification of animals was not attempted.

A benefit of the distance sampling survey technique, is that no catching and marking is required, meaning that it is a much less resource intensive and welfare friendly approach. Permanent marking, such as ear notches have been used for dog population estimates [33] but they require accurate measurement of the number of notched dogs released, which is not always possible in field situations. Beck, (1973) popularised a method requiring photographic comparison of dogs to identify individuals, rather than physical marking [15]. While this method is useful as it does not require resource intensive catching of dogs, in large scale operations it may be impractical to manually match photographs. Pattern recognition software is available for species such as wild dog and giraffe with characteristic coat patterns [34]. This would need to be validated for domestic dogs which have fewer discriminatory patterns to be used in large scale photographic surveys.

The distance sampling method assumptions include random placement of the survey lines which are, by definition, not valid when travelling along roads for the survey. Roads and human activity are likely to be associated with more dogs, resulting in an overestimation. We purposively subset the study area to within 100 m of known roads, to minimise the overestimation in areas unlikely to include dogs. The areas outside of the 100 m buffer were visually confirmed on satellite images to be areas with few to no buildings. Despite this, our estimates for the distance sampling methods may have reflected this overestimation and were, on average per working area, higher than the Chapman mark-resight methods. When considering estimates for the entire study area, however, there was overlap in the confidence intervals between methods. Another limitation of the distance sampling method, is that we did not take group size into account in the final analysis due to data reliability issues. Dogs were often seen roaming in groups and this may have affected the estimates.

There is added complexity to these surveys as many owned dogs may have variable roaming patterns. For example, dogs may be restricted during the time of marking (vaccination) and therefore not taken into account as a marked free-roaming dog, and then later allowed to roam freely during the resight surveys and considered free-roaming. It is unknown in what direction this may affect estimates, and household surveys may help address this. As this study only looked at free-roaming dogs, a household survey would be needed in any case for a complete picture of the dog population.

This study did not explicitly look at the interpretation of the marked proportion of dogs as vaccination coverage. However, the data would suggest that the vaccination coverage in this population is lower than the targeted figure of 70%. As part of the campaign, additional follow-up vaccination drives would have taken place in areas with lower coverage, which are not reflected in this study. Secondly, this study represents a subset of the dog population in the area, i.e. free-roaming dogs only, and does not take into account confined dogs. The vaccination coverage in unowned, free-roaming dogs may be lower than the general population due to difficulties in catching dogs that are not used to being handled but has not been routinely studied. This subset of the population may need alternative vaccination efforts, such as oral bait vaccine, to ensure adequate coverage.

Initial dog population estimates are important for planning the resources, time and funds needed for pilot dog vaccination or population management initiatives. However, this should be off-set against diverted limited resources that could be invested in the intervention. In areas where there is limited or no data about the dog population, the use of efficient survey methods enables initial population estimates to be calculated without detracting resources from the intervention. Data from initial pilot work can then be used to refine population estimates [12].

## Conclusion

In summary, the distance sampling method was adequately comparable to Chapman mark-resight in this study for the purpose of overall population estimates of free-roaming dogs across large areas containing multiple working zones, despite variation in abundance estimates within individual working zones. Although the distance sampling method requires more computational expertise, it could be valuable for resource limited control programmes if shown to give similar comparability in other regions over large areas. Systems which automate the computational components of estimating the wider population may make such methods more accessible in resource limited settings. Additionally, using only a representative number of roads to conduct the resight surveys may further save resources with an acceptable level of uncertainty in the population abundance estimation.

## Additional files

Additional file 1: **Tables S1.** Final detection function models used to estimate dog density and abundance using the Distance package in R. (DOCX 12 kb)

Additional file 2: **Figure S1.** Bland-Altman plots are given for the difference between Chapman and distance-method estimates, as well as the difference between methods for all roads and the subset of roads. (DOCX 177 kb)

## Data Availability

The datasets used during the current study are available from the corresponding author on reasonable request and with the permission of Mission Rabies. Map data is adapted from GADM for Fig. 1. https://gadm.org. The cartography in Fig. 2 has been adapted from OpenStreetMap under the Creative Commons Attribution-ShareAlike 2.0 license (CC BY-SA). https://www.openstreetmap.org/copyright

## Abbreviations

AIC: Akaike information criterion
CCC: Concordance correlation coefficient
GPS: Global Positioning System
WVS: Worldwide Veterinary Services
