# Supplementary material for: A comparison of population estimation techniques for individually unidentifiable free-roaming dogs
Source: BMC Vet Res. 2019 Jun 7;15:190. doi: 10.1186/s12917-019-1938-1 (PMC6556045; doi:10.1186/s12917-019-1938-1)
Supplement: Supplementary file 1 — Tables S1. Final detection function models used to estimate dog density and abundance using the Distance package in R. (DOCX 12 kb) [file 12917_2019_1938_MOESM1_ESM.docx]

|  | **Key function** | **Adjustment terms** | **Formula** |
| --- | --- | --- | --- |
| **All roads** | Half-normal | Cosine, order 4 | ~1 |
| Survey 1 | Half-normal | Cosine, order 2 | ~1 |
| Survey 2 | Hazard- rate | - | ~ time of day |
|  |  |  |  |
| **Subset roads** | Hazard- rate | - | ~ time of day + rural/urban |
| Survey 1 | Hazard- rate | - | ~surveyor |
| Survey 2 | Hazard- rate | - | ~1 |

***Additional Table 1:*** *Final detection function models used to estimate dog density and abundance using the Distance package in R. Regions were classified as working zones for all models.*
