# Supplementary material for: A comparison of population estimation techniques for individually unidentifiable free-roaming dogs
Source: BMC Vet Res. 2019 Jun 7;15:190. doi: 10.1186/s12917-019-1938-1 (PMC6556045; doi:10.1186/s12917-019-1938-1)
Supplement: Supplementary file 2 — Figure S1. Bland-Altman plots are given for the difference between Chapman and distance-method estimates, as well as the difference between methods for all roads and the subset of roads. (DOCX 177 kb) [file 12917_2019_1938_MOESM2_ESM.docx]

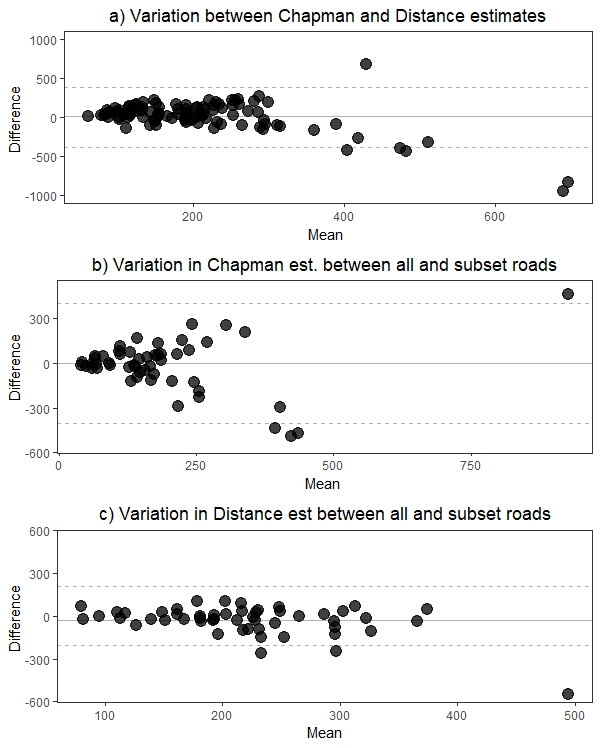
***Additional Figure 1****: Bland-Altman plots are given for the a) difference between Chapman and distance-method estimates, b) and c) difference between estimates for all roads and subset of roads. The mean of the estimates (x-axis) are plotted against the difference between estimates (y-axis). The mean of the difference is shown by the horizontal solid grey line and 2*SD given by the dashed lines.*
